# Associations of takeaway outlets with takeaway food consumption and adiposity: longitudinal analysis of the Fenland cohort

**DOI:** 10.1002/oby.24152

**Published:** 2024-10-09

**Authors:** Jody C. Hoenink, Thomas Burgoine, Nita G. Forouhi, Pablo Monsivais, Stephen J. Sharp, Jenna Panter, Jean Adams

**Affiliations:** ^1^ MRC Epidemiology Unit University of Cambridge School of Clinical Medicine, Institute of Metabolic Science Cambridge UK; ^2^ Elson S. Floyd College of Medicine Washington State University Spokane Washington USA

## Abstract

**Objective:**

This study builds on prior findings that link increased availability of takeaway food outlets in home, workplace, and commuting environments to greater takeaway consumption and adiposity. Using longitudinal data, we examine associations of takeaway availability at baseline with changes in consumption and adiposity between baseline and follow‐up.

**Methods:**

We analyzed data from the Fenland Study, with baseline data from 2005 to 2015 and follow‐up from 2015 to 2020. Takeaway outlet availability within 1 mile of participants' home and workplace addresses, based on 2011 local authority data, was assessed. Outcomes included takeaway food consumption (from a food frequency questionnaire) and body fat percentage (measured via dual‐energy x‐ray absorptiometry) at follow‐up.

**Results:**

Among 7581 participants (mean [SD] age, 49.3 [7.4] years) with a mean follow‐up of 6.7 years, no positive association was found between takeaway outlet availability at baseline and changes in consumption or body fat percentage. However, among the 12 associations tested, the highest combined home–workplace availability of takeaway outlets, compared with none, was associated with a 0.68 decrease in body fat percentage (95% CI: 0.24–1.12).

**Conclusions:**

Although takeaway outlet availability was linked to greater consumption and adiposity at baseline, it did not predict changes over time, underscoring the complexity of dietary behaviors and their relationship with the neighborhood food environment.

**Study Importance**

**What is already known?**

- There is inconsistent evidence on the associations between takeaway food outlet availability and dietary behaviors and adiposity.
- Most previous studies have used cross‐sectional analyses and focused on takeaway outlet availability in the residential neighborhood, neglecting other environments such as workplaces.

**What does this study add?**

- Using prospective cohort data, we found positive cross‐sectional associations of baseline takeaway outlet availability with takeaway consumption and adiposity. These positive findings were replicated at follow‐up.
- Using longitudinal data, we found that baseline takeaway outlet availability did not predict changes in consumption or adiposity over time. This was modified by age; there was evidence of some association of takeaway outlet availability with body fat change in younger adults (age <50 years).

**How might these results change the direction of research?**

- Future research should make further use of longitudinal designs, avoiding healthy cohort effects and maximizing study power.
- Given that takeaway food tends to be energy‐dense and nutrient‐poor, with known associations among consumption, dietary quality, and body weight, efforts to create healthier food environments may still be important public health measures.

## INTRODUCTION

The food environment plays a role in shaping people's behaviors and decisions, including their dietary choices [1]. One aspect of the food environment is the presence of food outlets in our neighborhoods, which determines the types of foods accessible for purchase and consumption. Among these food outlets, hot food takeaway outlets (hereafter referred to as “takeaways”) stand out as providing energy‐dense and nutrient‐poor foods, potentially leading to less healthy dietary behaviors and increased body weight [2].

Evidence linking food outlet exposure to diet and health outcomes has been mixed [3, 4]. For example, in our prior study conducted in the UK using cross‐sectional data from the Fenland Study, we found positive associations between availability of takeaways in residential neighborhoods (i.e., the count of takeaways within a buffer around the home) and takeaway food consumption and body weight [5]. In contrast, other UK‐based studies have not observed cross‐sectional associations between the residential food environment and these outcomes [6, 7]. Some sources of inconsistency that may explain these mixed findings include differences in measures of the food environment (e.g., proximity vs. count), outcomes (e.g., fruit and vegetable intake vs. dietary quality), and context (e.g., urban vs. rural) [4, 8].

Historically, most food environment research has concentrated on the residential food environment [4, 9], thereby neglecting the significance of other settings where individuals might regularly encounter food outlets [10]. Studies from the United States and the UK have found that takeaway availability was twice as high around the workplace than around the home [11, 12]. Furthermore, our previous work demonstrated that accounting for takeaway availability around the home, workplace, and commuting routes yielded stronger cross‐sectional associations with takeaway food consumption and body weight relative to solely including availability around the home [5]. A recent Dutch study found that takeaway availability around combined home–workplace settings was strongly correlated with total time‐weighted takeaway availability [13], suggesting that home and workplace settings may capture a large portion of an individual's exposure to takeaways.

Although food environment research across multiple domains such as the home, workplace, and commuting routes marks a significant methodological development, the predominance of cross‐sectional analyses within existing studies emerges as a notable limitation [4]. Cross‐sectional analyses are limited in their ability to account for self‐selection bias to permit robust causal inference or effectively track temporal changes in exposure and outcomes [9]. This shortcoming is particularly relevant when considering the Bradford Hill criteria for causation [14], which emphasize the importance of temporality in establishing a causal relationship. In cross‐sectional analyses, the temporal sequence between exposure and outcome is ambiguous, undermining the strength of causal evidence.

Our study sought to address this gap by transitioning from cross‐sectional to longitudinal analyses. By doing so, we ensure that the exposure to takeaway outlets precedes observed changes in dietary behaviors and adiposity. Therefore, this study set out to explore the associations of takeaway availability around the home and workplace at baseline with changes in takeaway food consumption and adiposity from baseline to follow‐up among adults from the Fenland Study.

## METHODS

We performed a longitudinal analysis to examine the relationships of takeaway outlet availability around the home and workplace at baseline (T0) with changes in takeaway food consumption and body fat percentage between T0 and follow‐up (T1) using data from the Fenland Study. The Fenland Study aims to assess how genetic, behavioral, environmental, and social factors influence diabetes, obesity, and related metabolic disorders [15]. Our methodological approach, aligning with our previous study [5], ensures consistency in exposure and outcome measures but introduces some modifications in covariate adjustments and adopts body fat percentage as a more accurate measure of adiposity compared with the previously used body mass index (BMI), which was used as a secondary outcome measure.

### Study sample

**FIGURE 1:**
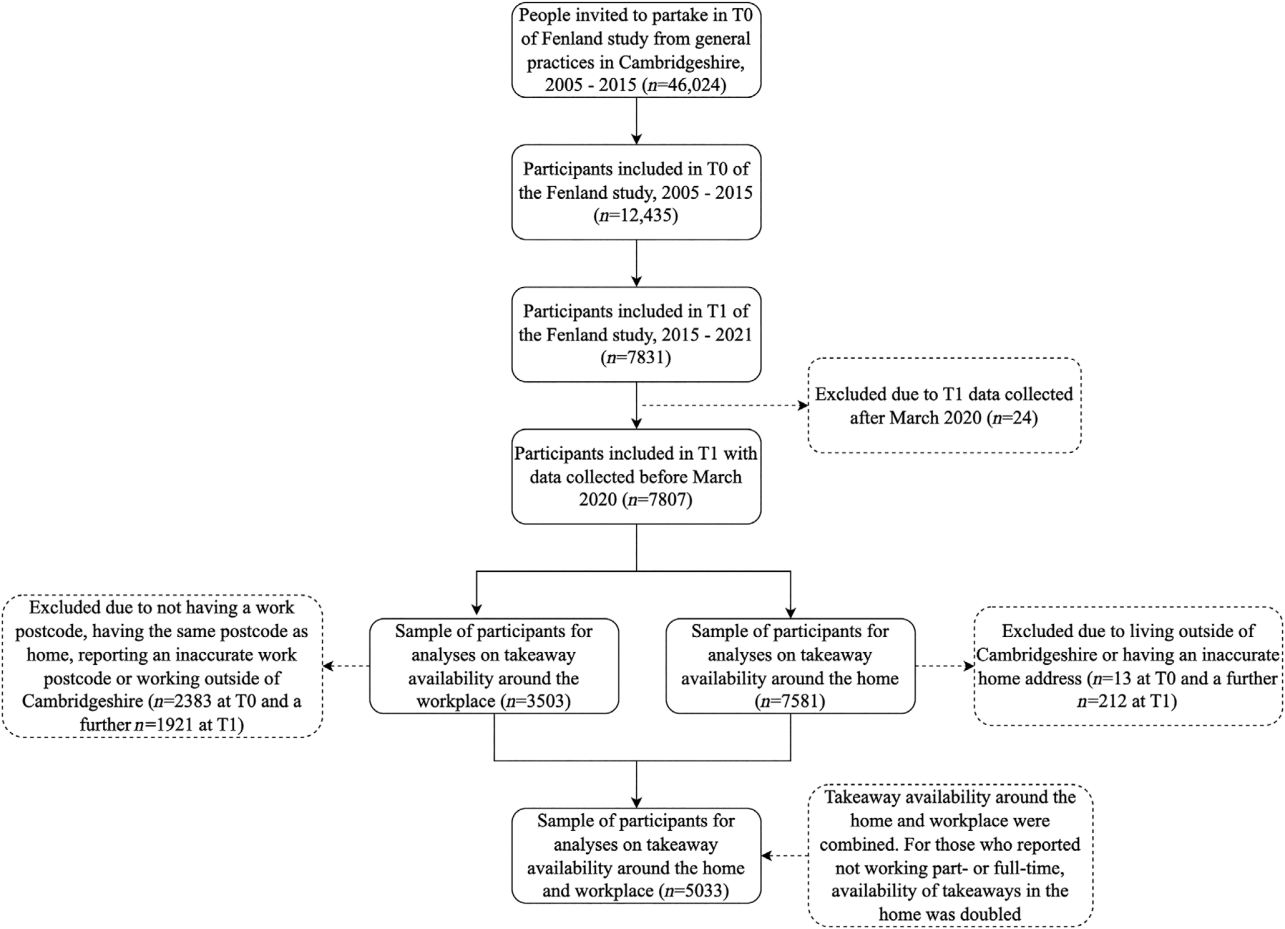
Flowchart of participant inclusion.

The Fenland Study initially recruited 12,435 adults (response rate, 27%), who were born between 1950 and 1975 in Cambridgeshire, UK, from 2005 to 2015, with follow‐up data collected from 7831 participants between 2015 and 2021 (37% of participants lost to follow‐up; Figure 1). Participants, recruited from general practice lists in Cambridge, Ely, and Wisbech, completed lifestyle questionnaires and a semiquantitative food frequency questionnaire (FFQ) at both time points. The study was approved by the Health Research Authority National Research Ethics Service Committee East of England‐Cambridge Central. The study complies with the Declaration of Helsinki and informed consent was obtained from all the participants before starting the study.

We excluded participants without follow‐up data or data collected after March 2020 (due to the COVID‐19 pandemic onset in the UK). For home‐focused analyses, participants living outside Cambridgeshire were excluded, and, for workplace analyses, those working outside Cambridgeshire or without a fixed workplace address were also excluded.

### Dependent variables

Our primary outcomes were as follows: 1) consumption of energy‐dense takeaway foods; and 2) body fat percentage, selected based on the association with takeaway food consumption's proximal impact and its linkage to increased body weight and adiposity [15, 16].

#### Assessment of takeaway food consumption

Takeaway food consumption was quantified in grams per day of pizza, burgers, fried fish, and French fries. These quantities were calculated using the FFQ EPIC Tool for Analysis (FETA) tool [17] based on participants' self‐reports of portion sizes from a self‐administered, 130‐item, semiquantitative FFQ at T0 and T1. This measurement, validated against self‐reported takeaway meal frequency, served as a marker of takeaway‐type food consumption.

#### Assessment of body fat percentage

Body fat percentage was determined using bioelectrical impedance analysis over dual‐energy x‐ray absorptiometry at both time points due to greater data availability. Participants attended a clinical research facility after an overnight fast, where trained research assistants measured their height (centimeters) and weight (kilograms). Body fat percentage as a measure of adiposity was preferred over the widely and previously used measure BMI [5] due to its direct assessment of body fat and greater accuracy in obesity classification [18]. BMI and fat mass index (FMI) were calculated as secondary and alternative adiposity measures. BMI was calculated as weight (kilograms) divided by height (meters squared), and FMI was calculated as fat mass (kilograms; measured using dual‐energy x‐ray absorptiometry) divided by height (meters squared).

### Independent variables

Takeaways were considered to be retail establishments that primarily offer hot food designed for consumption off the premises and where orders are placed and paid for at the counter [15]. They typically have no wait staff, provide limited or no dine‐in options, and encompass both chain and independent outlets. Takeaway data were collected from 10 local authorities covering the study area in 2011 (T0) and 2017 (T1). In the UK, it is mandatory for food outlet owners to register their establishments with the local council and inform the council if they cease operations, resulting in local councils possessing up‐to‐date records of food outlets, which are regarded as the most reliable source of data on food outlets in the UK [19]. Previous research has validated the use of these secondary data sources in food environment research and has described the methodology for defining takeaways in detail [5, 12]. Participants' home and workplace addresses at T0 and T1 were mapped by postcode using a geographic information system (GIS; ArcGIS Pro 3.0, Esri, Inc., Redlands, California).

Similar to our previous studies in this cohort [5, 15], takeaway availability was defined as a 1‐mile euclidean buffer from the centroid of a participant's home or workplace postcode, as previous evidence has suggested that this definition relates closely to actual food purchasing behaviors among UK adults [20]. Takeaway availability in combined home and workplace neighborhoods was calculated by summing availability around the home and workplace. For participants who reported not working either part or full time (e.g., because they were keeping house or retired), availability of takeaways in the home setting was doubled.

Given the nonlinear associations with outcomes, we categorized takeaway availability into three groups. The first group included participants with zero takeaway availability, whereas the subsequent groups were based on tertile distributions of the remaining participants at T0. Because we determined that takeaway availability at T0 and T1 were very similar (Tables S1 and S2), suggesting that very little change in takeaway availability has taken place, longitudinal analyses only included takeaway availability data at T0.

### Covariates

Individual‐level covariates measured at T0 were sex (male or female), age (in years), age at completion of full‐time education (in years), annual household income (<£20,000, £20,000–£39,999, or ≥£40,000) and occupational social class (professional, intermediate, or working class) [15]. Different from our previous study [5], we also adjusted for current work status (eight response options, i.e., full‐time work, part‐time work, keeping house or carer, retired, unemployed, waiting to start a new job, temporarily sick, or permanently sick). To control for residential neighborhood self‐selection during the study period, relocation status was calculated by determining whether a participant moved between residences from T0 to T1 using the home postcode. Furthermore, we calculated the follow‐up time in years between measurements (date measurement T1–date measurement T0).

We considered two further area‐level covariates that could potentially influence our independent and dependent variables. We included supermarket availability at T0 (i.e., large chain supermarkets such as Tesco and Sainsbury's), measured the same way as described for takeaway availability. Different from our previous study [5], we additionally included quintiles of the relative rank of Index of Multiple Deprivation (IMD) 2010 scores for home addresses at T0, which is a measure of deprivation at the lower super output area (i.e., small areas designed to be of a similar population size with an average of ~1500 residents) level [21].

### Statistical analysis

We report sample characteristics at T0 and T1 using the mean (SD) for symmetrically distributed continuous variables, median (interquartile range [IQR]) for skewed continuous variables, and *n* (%) for categorical variables. For the outcome measures, i.e., takeaway food consumption and body fat percentage, we recoded outliers as the mean ± 3 SD.

We used linear regression models to estimate and test the associations of takeaway availability in home and workplace neighborhoods at T0 with takeaway food consumption and body fat percentage at T1 adjusted for these outcomes at T0. This resulted in a total of six separate analyses with three exposure measures, two outcome measures, and six different sample sizes. All models were adjusted for baseline measurements of age, sex, age at completion of full‐time education, work status, annual household income, occupational social class, IMD quintiles (not for models including takeaway availability around the workplace), and supermarket availability. Models were also adjusted for follow‐up time and home relocation during the study period. Individuals with missing values were excluded (i.e., a complete‐case analysis).

Given the large sample size and small intraclass correlation coefficient of 0.015 or less (i.e., between‐group variance/[between‐group variance + within‐group variance]), models were not adjusted for clustering of participants within postcodes. Statistical significance was set at a two‐sided α level of 0.05, and all analyses were conducted in R version 4.2.1 (R Project for Statistical Computing, Vienna, Austria).

### Sensitivity analyses

Several sensitivity analyses were run to assess the robustness of our findings. First, to assess our findings' sensitivity to the categorization choice, we divided takeaway availability into four rather than three groups. Second, as reported earlier, analyses with takeaway availability in 1‐mile buffers were replicated with two additional measures of adiposity (i.e., BMI and FMI). Third, reflecting the current uncertainty in food environment research regarding the buffer size of these home and workplace neighborhoods [22, 23], we included other common definitions of takeaway exposure. Thus, we calculated takeaway availability in 400‐m and 800‐m radius buffers and street‐network proximity (meters) to takeaways as the distance from the centroid of a participant's home or workplace postcode to the following: 1) the nearest takeaway; and 2) the average distance to the five nearest takeaways [22].

## RESULTS

The overall sample was *N* = 7581, and missing data ranged from 0% (e.g., sex) to 5% (i.e., IMD). Mean (SD) age of participants at T0 was 49.3 (7.4) years (Table 1), with a mean time of 6.7 years between measurements. At T0, 25% and 19% of participants had no takeaways available in 1‐mile buffers around the home and workplace, respectively (Table 1). Median takeaway food consumption was 1.5 servings per week (IQR, 1.0–2.5) at both T0 and T1. Mean (SD) body fat percentage at T0 was 32.7% (8.4%) and 33.3% (8.7%) at T1. Compared with participants with data at both time points, participants who were lost to follow‐up were more often female, younger, working class, had a household income of <£20,000, and had a higher mean body fat percentage (Table S3).

**TABLE 1:** Characteristics of the Fenland Study sample (*n* = 7581) at T0.

		*n*	Summary statistics
Age, y, mean (SD)		7581	49.3 (7.4)
Female, *n* (%)		7581	3924 (51.8)
Age at completion of full‐time education, y, mean (SD)		7529	19.3 (4.4)
Current work status, *n* (%)	Working full time (>30 h/wk)		5093 (67.6)
	Working part time (<30 h/wk)		1584 (21.0)
	Keeping house or carer		383 (5.1)
	Wholly retired from work	7532	283 (3.8)
	Unemployed or waiting to start new job		119 (1.6)
	Temporarily or permanently sick		70 (0.9)
Annual household income, *n* (%)	<£20,000		833 (11.2)
	£20,000–£39,999	7419	2518 (33.9)
	≥£40,000		4068 (54.8)
Occupational social class, *n* (%)	Working class		1407 (19.4)
	Intermediate	7267	1228 (16.9)
	Professional		4632 (63.7)
IMD score in quintiles, mean (SD)	1		3.8 (1.2)
	2		7.4 (1.4)
	3	7195	12.2 (1.5)
	4		18.4 (2.1)
	5		28.2 (6.1)
Takeaway food consumption, g/d, median (IQR)		7557	30.0 (14.0–46.8)
Body fat percentage, mean (SD)		7535	32.7 (8.4)
FMI, mean (SD)		7310	0.9 (0.3)
BMI, mean (SD)		7580	26.5 (4.4)
Takeaways around the home (minimum–maximum)	No takeaways	1863	0–0
	Some takeaways	2960	1–8
	Most takeaways	2758	9–49
Takeaways around the workplace (minimum–maximum)	No takeaways	632	0–0
	Some takeaways	1336	1–10
	Most takeaways	1429	11–62
Takeaways around the home and workplace (minimum–maximum)	No takeaways	605	0–0
	Some takeaways	2159	1–17
	Most takeaways	2269	18–103
Supermarkets around the home, median (IQR)		7581	1 (0–3)
Supermarkets around the workplace, median (IQR)		3397	2 (0–5)
Supermarkets around the home and workplace, median (IQR)		5033	3 (1–8)

Abbreviations: FMI, fat mass index; IMD, Index of Multiple Deprivation.

### Associations of takeaway availability at T0 with changes in takeaway consumption and body fat percentage (T0–T1)

**FIGURE 2:**
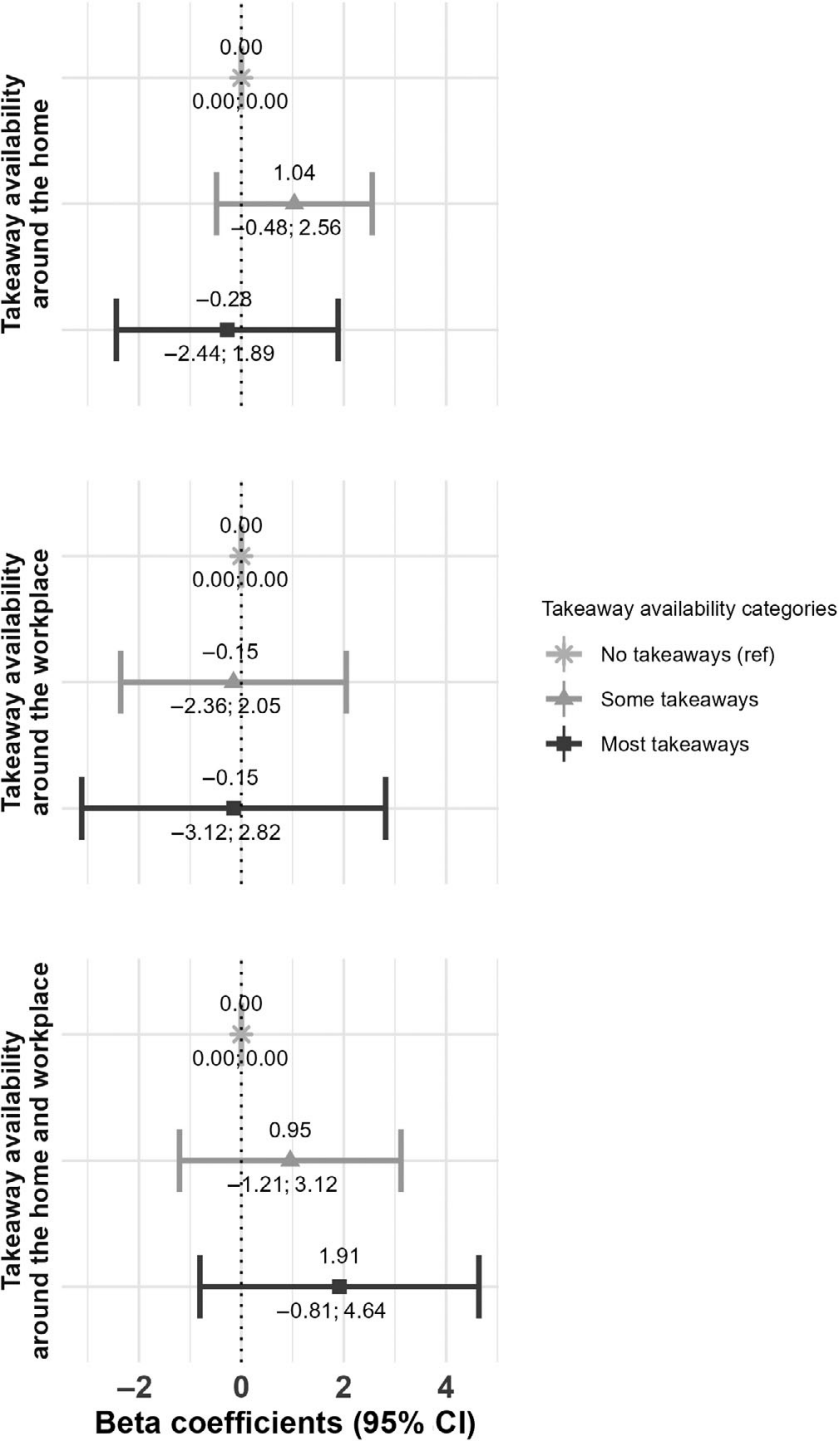
Associations between takeaway availability at T0 and takeaway consumption at T1. In the home, workplace, and combined environments, *n* = 6013, *n* = 2905, and *n* = 4008 participants were included, respectively. Associations were adjusted for baseline outcome, age, sex, age at completion of full‐time education, work status, annual household income, occupational social class, Index of Multiple Deprivation (IMD) quintiles (only for home and combined environments), and supermarket exposure, as well as follow‐up time and home relocation.

**FIGURE 3:**
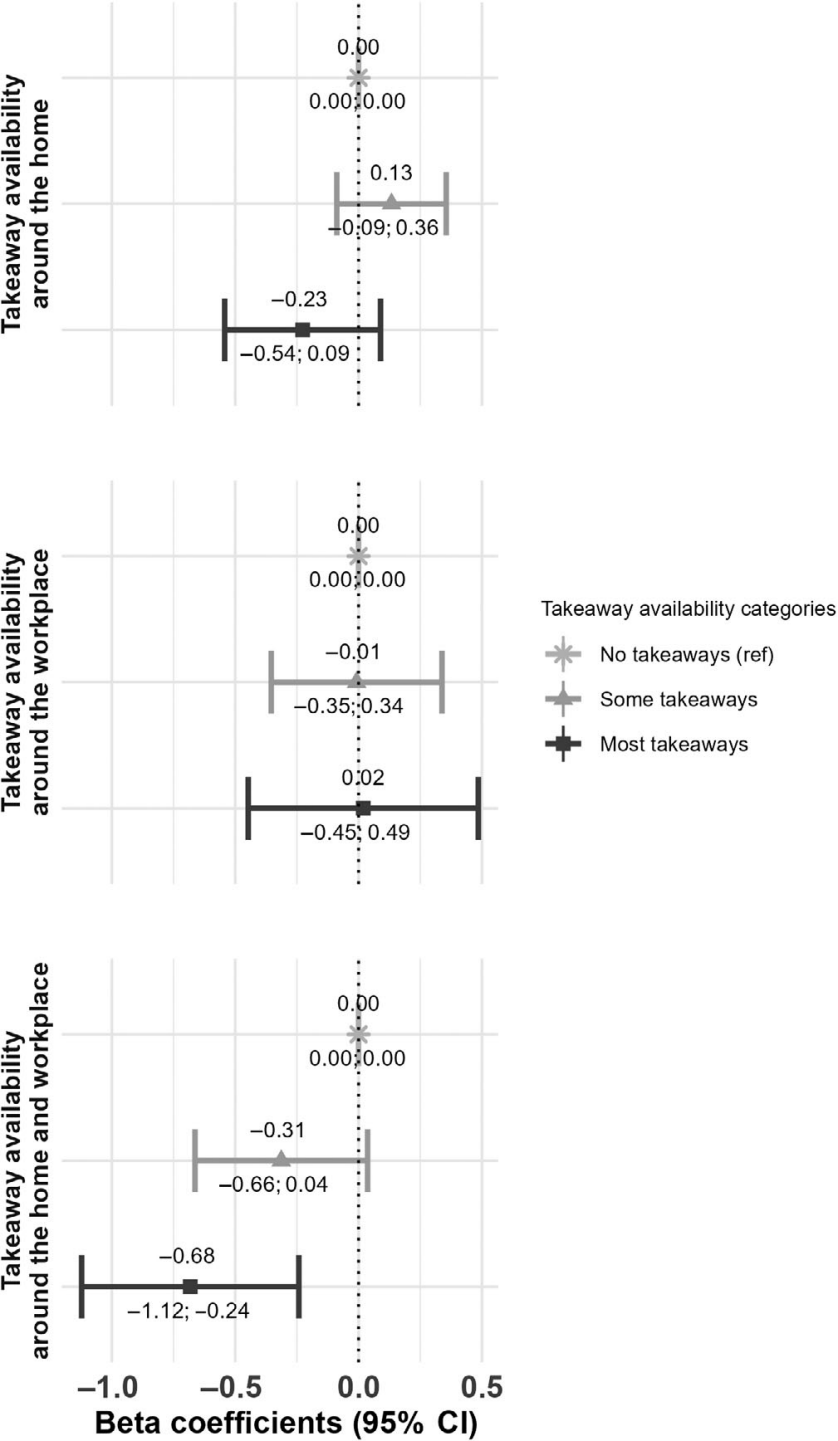
Associations between takeaway availability at T0 and body fat percentage at T1. In the home, workplace, and combined environments, *n* = 6670, *n* = 3216, and *n* = 4445 participants were included, respectively. Associations were adjusted for baseline outcome, age, sex, age at completion of full‐time education, work status, annual household income, occupational social class, Index of Multiple Deprivation (IMD) quintiles (only for home and combined environments), and supermarket exposure, as well as follow‐up time and home relocation.

We mostly observed no associations of availability of takeaways at T0 with changes in takeaway food consumption (Figure 2) or body fat percentage from T0 to T1 (Figure 3). Only when considering the availability of takeaways at T0 around combined home and workplace neighborhoods, a negative association was observed; compared with those with no takeaway availability, those with high takeaway availability had a decreased body fat percentage from T0 to T1 (β_high takeaway availability_, −0.68; 95% confidence interval [CI]: −1.12 to −0.24; Figure 3). This finding was consistent for the outcome measures BMI and FMI (Table S4), but not takeaway consumption (Figure 2) or when considering other measures of takeaway exposure (Table S5). Sensitivity analyses supported the null findings.

### Additional analyses

In contrast to our earlier cross‐sectional findings within this cohort [5, 15], our analyses investigating takeaway availability at T0 and changes in outcomes from T0 to T1 did not demonstrate the same positive associations. To further investigate this discrepancy, we performed post hoc cross‐sectional analyses at T0 and T1 (Table S6). Cross‐sectional analyses revealed expected positive associations at both time points. Specifically, at T0, we observed positive associations between takeaway availability around the workplace and combined home and workplace neighborhoods and takeaway food consumption and body fat percentage. At T1, greater availability of takeaways around the home was associated with greater takeaway consumption. Also, greater availability of takeaways around the home, workplace, and combined was associated with greater body fat percentage.

Considering previous evidence suggesting that age may influence the association between baseline takeaway availability and adiposity changes [24], we examined age as a potential effect modifier. We found one statistically significant interaction: for adults under age 50 years, takeaway availability around the home was associated with a 0.32 (95% CI: 0.00–0.64) increase in body fat percentage from T0 to T1, with no association observed in those over age 50 years (Figure S1). Age did not modify the associations of takeaway availability around the workplace or in combined settings with changes in consumption or adiposity.

## DISCUSSION

In our sample from Cambridgeshire followed up over 7 years, we mostly found no evidence of associations of takeaway availability around the home and workplace at baseline with subsequent changes in takeaway food consumption and adiposity from T0 to T1. One out of twelve tested associations was observed: greater takeaway availability in combined home and workplace neighborhoods at baseline was associated with negative change in body fat percentage from T0 to T1. The mostly null findings were robust to replication using a variety of other takeaway exposure and adiposity measures. In additional analyses, we replicated our prior results within this cohort [5, 15], observing positive cross‐sectional associations of takeaway availability with both takeaway consumption and adiposity at T0 and now also at T1.

The general lack of associations of takeaway availability with changes in takeaway food consumption and adiposity over time aligns with other longitudinal food environment research. A scoping review exploring longitudinal associations between residential neighborhood characteristics and obesity revealed that 39% of studies observed associations in the expected direction when examining fixed neighborhood characteristics against varying obesity outcomes [9]. Nearly half of the studies (46%, *n* = 223) assessed indicators related to the food environment, and associations were less consistent for the food environment compared with, e.g., socioeconomic attributes (second most prevalent neighborhood characteristic studied). Similar to our study, a previous study found some evidence of a negative association between baseline takeaway availability around the home and weight trajectories [25], although this finding is relatively uncommon in the field [9]. Given that this was the only statistically significant result among the 12 associations that we examined, and it was mostly not replicated in sensitivity analyses, our finding may merely be a chance occurrence.

Our study's contrasting results, with no longitudinal associations but positive cross‐sectional ones, echo findings from similar research such as a US study of over 100,000 participants that linked residential environmental factors, including takeaway availability, to baseline weight, but not to 5‐year weight change [26]. A Dutch study also found stronger associations with initial BMI than with BMI change over 4 years, particularly in participants ages 40 to 49 years, with no associations in other age groups [24]. Our study also identified age as an effect modifier, with positive associations between takeaway availability and body fat change in adults under age 50 years, but not in those over age 50 years.

We hypothesized that increased takeaway availability would increase consumption and adiposity over time, but our findings did not support this. The initial increase in takeaway food consumption among individuals in high‐availability areas, as suggested by our cross‐sectional findings, did not persist, possibly due to ceiling effects on consumption influenced by factors such as meal frequency, affordability, and alternative food preferences, leading to stable dietary behaviors over time [27].

Despite the link between habitual overconsumption of takeaway food and weight gain, we did not observe a corresponding increase in adiposity over time [28]. This absence of a detectable increase might be explained by the short time lag between overconsumption and weight gain. Despite our research not demonstrating such increases in adiposity over time, it is important to acknowledge that adiposity is affected by a complex interplay of factors beyond just one type of food consumption, which may reduce the detectability of an association with baseline takeaway availability. It might be that *change* in takeaway availability is associated with change in adiposity, but it was not possible for us to study this.

Our analysis focused solely on baseline takeaway availability due to minimal change in takeaway numbers. We found a near‐equal distribution of participants across availability groups from T0 to T1, with over 70% experiencing no change in takeaway availability. The number of takeaway outlets increased by only 6% over 6 years, from 731 in 2011 to 776 in 2017. This growth rate, projected linearly, suggests an 18% increase over 18 years, which is slower compared with Norfolk's 45% rise between 1990 and 2008 [29]. Further research could compare this trend, potentially looking at more recent data and examining individuals who experience more rapid environmental changes, such as recent movers, to better understand how shifts in takeaway availability might influence dietary behaviors and obesity.

Our null findings in longitudinal versus cross‐sectional models may be attributed to a lack of power, with longitudinal models showing smaller effect sizes due to modest increases in adiposity and stable takeaway food consumption over time. This minimal change points to a “healthy cohort effect,” in which participants are generally healthier or more health‐conscious than the general population. For instance, those who remained in the study had a baseline body fat percentage of 32.7%, which is lower than the 34.6% of those who dropped out. Contrasting our results, a New Zealand study observed a greater BMI increase and positive links between takeaway proximity change and BMI [22]. We also noted a positive association between takeaway availability and body fat change in younger adults, with a mean change of 1.1 points versus 0.5 in older adults. Future research could use routinely collected data to reduce the “healthy cohort effect” and reflect broader population impacts more accurately. Extended follow‐up periods or larger sample sizes might also reveal more significant outcome changes.

Our longitudinal analyses did not demonstrate a link between baseline takeaway availability and changes in takeaway food consumption and adiposity over time, indicating that limiting the availability of takeaways may not be sufficient to promote healthier dietary behaviors and reduce adiposity. Nonetheless, we believe that these findings should not be used as an argument against the implementation of policy measures aimed at improving the food environment. The potential underestimation of the obesogenic influence of takeaways, due to limitations in study power, underscores the need for caution in interpreting our results. The persisting cross‐sectional associations observed at both baseline and follow‐up underscore the possibility that interventions targeting takeaway availability may still be beneficial. Takeaway food is generally characterized by lower nutritional value and higher caloric content [16], contributing to increased adiposity when consumed frequently [30]. Policies aimed at reducing takeaway availability, particularly in socioeconomically disadvantaged neighborhoods where they are disproportionately concentrated [29], may still hold promise for reducing obesity prevalence and mitigating its associated social and health inequities. Although acknowledging the complex interplay of many and varied factors contributing to diet‐related illnesses, environmental interventions may be one component of a comprehensive public health strategy to promote healthier behaviors and prevent diet‐related disease and ill health.

Using routinely collected data and an extended follow‐up period to improve power will also allow for the identification of how takeaway availability may interact with modifying factors such as age and genetics on adiposity over the life course [24]. Future studies should also explore the online food environment and adopt more nuanced methods for measuring physical food outlet exposure. The growth of online food delivery services may have altered the impact of physical food outlets on dietary behaviors [31], potentially diminishing their influence on dietary habits and change in adiposity. Traditional GIS‐derived metrics do not fully capture the complexity of the food environment's impact on obesity due to their static nature and disregard for actual human movement within geographic boundaries [32]. More dynamic, person‐centric methods such as ecological momentary assessments, global positioning system (GPS) tracking, and self‐reported activity diaries could offer a clearer view of individual interactions with the food environment [10, 13, 33, 34]. Incorporating actual use data, exposure duration, and household roles in food purchasing may further refine these exposure measurements [34, 35, 36].

Our study has several strengths, including longitudinal data and using body fat percentage for a more precise adiposity measure over BMI, which cannot distinguish fat from muscle [37]. We evaluated takeaway availability around the home and workplace for a more thorough availability assessment and used accurate, legally mandated local authority data for takeaway locations. However, our focus was limited to takeaways and supermarkets, omitting other food outlets such as restaurants and ethnic markets that might also affect diet and adiposity. Although adjusting for relocation status mitigated some bias, our findings might still be influenced by neighborhood self‐selection. The temporal discrepancy between takeaway data (2011) and outcomes (2005–2015 and 2015–2020) probably had minimal impact due to the relatively stable takeaway availability. Our sample reflects the regional demographic but may not fully represent other UK areas or the national demographic [15]. Additionally, the generalizability of our results may further be constrained by differential loss to follow‐up. Notably, a higher proportion of female individuals and individuals with a lower socioeconomic position were lost to follow‐up compared with those who remained in the study. Last, the stronger association in younger adults suggests that the middle‐aged cohort might not capture those most vulnerable to takeaway availability's effects. This, along with the negligible change in takeaway availability and limited outcome variance, prompts further reflection on the cohort's suitability for exploring diet and adiposity changes linked to takeaway availability.

## CONCLUSION

In this UK study, we explored associations of takeaway availability around the home and workplace with changes in takeaway food consumption and adiposity over a 7‐year period. We observed predominantly null results, which overall indicated no evidence of associations in these relationships. This contrasts with our cross‐sectional results and findings from previous research in this cohort, which showed positive associations between takeaway availability and takeaway consumption and adiposity at both baseline and follow‐up. Our findings suggest that although people living in areas with more takeaways have greater takeaway consumption and body fat percentage, this does not necessarily lead to an increase in these outcomes over time. Future research should explore the relationship between changes in the food environment and adiposity using study designs that harness changes in exposure and outcome measures. Such studies should use population‐representative samples and person‐centric methods for measuring food outlet exposure.

## FUNDING INFORMATION

The Fenland Study is funded by the Medical Research Council (MRC), and the study principal investigators (Nita G. Forouhi) acknowledge this support (grant numbers MC_UU_00006/1, MC_UU_00006/3, and MC_UU_00006/6). Jody C. Hoenink, Jenna Panter, Jean Adams, and Thomas Burgoine are currently supported by the MRC (unit program number MC_UU_00006/7). Pablo Monsivais received support from the Health Equity Research Center at Washington State University. Nita G. Forouhi acknowledges support from the National Institute of Health Research (NIHR) Cambridge Biomedical Research Centre (BRC) Nutrition, Obesity, Metabolism and Endocrinology Research Theme (NIHR203312). Nita G. Forouhi is an NIHR Senior Investigator (NIHR202397). The views expressed are those of the authors and not necessarily those of the NIHR or the Department of Health and Social Care. The funders played no role in the design of the study; the collection, analysis, and interpretation of data; or the writing of the manuscript.

## CONFLICT OF INTEREST STATEMENT

The authors declared no conflict of interest.

## Supporting information


**Data S1.** Supporting Information.
