# Supplementary material for: Associations of takeaway outlets with takeaway food consumption and adiposity: longitudinal analysis of the Fenland cohort
Source: Obesity (Silver Spring). 2024 Oct 9;32(12):2388–97. doi: 10.1002/oby.24152 (PMC11589533; doi:10.1002/oby.24152)
Supplement: Supplementary file 1 — Data S1. Supporting Information. [file OBY-32-2388-s001.docx]

**Supplementary Material**

Table S1. Takeaway and supermarket availability and accessibility in Fenland T0 and T1

|  | | T0 (2005-2015) | | T1 (2015-2020) | |
| --- | --- | --- | --- | --- | --- |
|  | | Participants (%) | Summary statistics | Participants (%) | Summary statistics |
|  | Home environment | | | | |
| Takeaways in 400m buffer around home environment (min-max) | No takeaways | 5041 (66) | 0 - 0 | 5073 (67) | 0 - 0 |
|  | Some takeaways | 1111 (15) | 1 – 1 | 998 (13) | 1 – 1 |
|  | Most takeaways | 1492 (19) | 2 – 17 | 1510 (20) | 2 – 21 |
| Takeaways in 800m buffer around home environment (min-max) | No takeaways | 2981 (39) | 0 – 0 | 2975 (39) | 0 – 0 |
|  | Some takeaways | 2272 (30) | 1 – 2 | 2222 (29) | 1 – 2 |
|  | Most takeaways | 2328 (31) | 3 – 27 | 2384 (31) | 3 – 34 |
| Takeaways in 1mile buffer around home environment (min-max) | No takeaways | 1863 (25) | 0 – 0 | 1986 (26) | 0 – 0 |
|  | Some takeaways | 2960 (39) | 1 – 8 | 2932 (39) | 1 – 8 |
|  | Most takeaways | 2758 (36) | 9 – 49 | 2663 (35) | 9 – 62 |
| Distance (in m) to nearest takeaway around home environment (mean (SD)) | Closest distance | 2527 (33) | 353 (166) | 2537 (33) | 360 (163) |
|  | Moderate distance | 2527 (33) | 917 (194) | 2503 (33) | 913 (198) |
|  | Furthest distance | 2527 (33) | 3568 (2539) | 2541 (34) | 4613 (3940) |
| Average distance (in m) to 5 nearest takeaways around home environment (mean (SD)) | Closest distance | 2528 (33) | 758 (289) | 2653 (35) | 743 (290) |
|  | Moderate distance | 2526 (33) | 2544 (976) | 2440 (32) | 2603 (959) |
|  | Furthest distance | 2527 (33) | 5927 (2330) | 2488 (33) | 6388 (3668) |
| Supermarkets in 400m buffer around home environment (median (p25-p75)) | | 7581 (100) | 0 (0 – 0) | 7581 (100) | 0 (0 – 0) |
| Supermarkets in 800m buffer around home environment (median (p25-p75)) | | 7581 (100) | 0 (0 – 1) | 7581 (100) | 0 (0 – 1) |
| Supermarkets in 1 mile buffer around home environment (median (p25-p75)) | | 7581 (100) | 1 (0 – 3) | 7581 (100) | 1 (0 – 3) |
| Distance (in m) to closest supermarket from home (median (p25-p75)) | | 7581 (100) | 1778  (808 – 5819) | 7581 (100) | 1862  (889 – 5898) |
| Average distance (in m) to 5 closest supermarkets from home (median (p25-p75)) | | 7581 (100) | 5482  (2123 – 8463) | 7581 (100) | 5574  (2380 – 8169) |
|  | Workplace environment | | | | |
| Takeaways in 400m buffer around work environment (min-max) | No takeaways | 2016 (59) | 0 – 0 | 1962 (58) | 0 – 0 |
|  | Some takeaways | 714 (21) | 1 – 2 | 714 (21) | 1 – 2 |
|  | Most takeaways | 667 (20) | 3 – 22 | 721 (21) | 3 – 26 |
| Takeaways in 800m buffer around work environment (min-max) | No takeaways | 1221 (36) | 0 – 0 | 1151 (34) | 0 – 0 |
|  | Some takeaways | 1040 (31) | 1 – 4 | 1086 (32) | 1 – 4 |
|  | Most takeaways | 1136 (33) | 5 – 33 | 1160 (34) | 5 – 39 |
| Takeaways in 1mile buffer around work environment (min-max) | No takeaways | 632 (19) | 0 – 0 | 601 (18) | 0 – 0 |
|  | Some takeaways | 1336 (39) | 1 – 10 | 1309 (38) | 1 – 10 |
|  | Most takeaways | 1429 (42) | 11 – 62 | 1487 (44) | 11 – 69 |
| Distance to nearest takeaway around work environment (mean (SD)) | Closest distance | 1132 (33) | 232 (147) | 1169 (34) | 221 (154) |
|  | Moderate distance | 1132 (33) | 876 (250) | 1129 (33) | 851 (232) |
|  | Furthest distance | 1132 (33) | 3638 (4381) | 1099 (32) | 4589 (11235) |
| Average distance to 5 nearest takeaways around work environment (mean (SD)) | Closest distance | 1134 (33) | 512 (230) | 1106 (33) | 486 (216) |
|  | Moderate distance | 1130 (33) | 1645 (402) | 1126 (33) | 1585 (369) |
|  | Furthest distance | 1132 (33) | 5613 (4528) | 1165 (34) | 6067 (11102) |
| Supermarkets in 400m buffer around work environment (median (p25-p75)) | | 3397 (100) | 0 (0 – 1) | 3397 (100) | 0 (0.– 0) |
| Supermarkets in 800m buffer around work environment (median (p25-p75)) | | 3397 (100) | 1 (0 – 2) | 3397 (100) | 0 (0 – 2) |
| Supermarkets in 1 mile buffer around work environment (median (p25-p75)) | | 3397 (100) | 2 (0 – 5.0) | 3397 (100) | 2 (0 – 4) |
| Distance (in km) to closest supermarket from work (median (p25-p75)) | | 3397 (100) | 1169  (557 – 2533) | 3397 (100) | 1394  (645 – 3376) |
| Average distance (in km) to 5 closest supermarkets from work (median (p25-p75)) | | 3397 (100) | 2817  (1464 – 6376) | 3397 (100) | 2732  (1718 – 6271) |
|  | Home + workplace environment | | | | |
| Takeaways in 400m buffer around home + workplace environment (min-max) | No takeaways | 2341 (47) | 0 – 0 | 2607 (52) | 0 – 0 |
|  | Some takeaways | 1235 (25) | 1 – 2 | 1051 (21) | 1 – 2 |
|  | Most takeaways | 1457 (29) | 3 – 34 | 1375 (27) | 3 – 42 |
| Takeaways in 800m buffer around home + workplace environment (min-max) | No takeaways | 1100 (22) | 0 – 0 | 1351 (27) | 0 – 0 |
|  | Some takeaways | 1978 (39) | 1 – 6 | 1814 (36) | 1 – 6 |
|  | Most takeaways | 1955 (39) | 7 – 48 | 1868 (37) | 7 – 68 |
| Takeaways in 1mile buffer around home + workplace environment (min-max) | No takeaways | 605 (12) | 0 – 0 | 847 (17) | 0 – 0 |
|  | Some takeaways | 2159 (43) | 1 – 17 | 2063 (41) | 1 – 17 |
|  | Most takeaways | 2269 (45) | 18 – 103 | 2123 (42) | 18 – 122 |
| Distance (in m) to nearest takeaway around home + workplace environment (mean (SD)) | Closest distance | 1678 (33) | 854 (341) | 1785 (35) | 856 (343) |
|  | Moderate distance | 1676 (33) | 2042 (436) | 1518 (30) | 2043 (445) |
|  | Furthest distance | 1678 (33) | 7453 (18606) | 1730 (35) | 19791 (68744) |
| Average distance (in m) to 5 nearest takeaways around home + workplace environment (mean (SD)) | Closest distance | 1677 (33) | 1760 (630) | 1744 (35) | 1700 (644) |
|  | Moderate distance | 1678 (33) | 4726 (1161) | 1482 (29) | 4742 (1191) |
|  | Furthest distance | 1677 (33) | 11504 (18554) | 1807 (36) | 22340 (67307) |
| Supermarkets in 400m buffer around home + workplace environment (median (p25-p75)) | | 5033 (100) | 0 (0 – 1) | 5033 (100) | 0 (0 – 1) |
| Supermarkets in 800m buffer around home + workplace environment (median (p25-p75)) | | 5033 (100) | 1 (0 – 3) | 5033 (100) | 1 (0 – 2) |
| Supermarkets in 1 mile buffer around home + workplace environment (median (p25-p75)) | | 5033 (100) | 3 (1 – 8) | 5033 (100) | 2 (0 – 7) |
| Distance (in m) to closest supermarket from home + workplace (median (p25-p75)) | | 5033 (100) | 3668  (1861 - 9118) | 5033 (100) | 3985  (1990 – 10121) |
| Average distance (in m) to 5 closest supermarkets from home + workplace (median (p25-p75)) | | 5033 (100) | 9277  (4621 – 14474) | 5033 (100) | 9472  (4975 – 14469) |

Table S2. Takeaway availability change around the home, workplace and home + workplace between T0 and T1

|  | *-2 change in takeaway availability between T0 and T1 (%)* | *-1 change in takeaway availability between T0 and T1 (%)* | *No change in takeaway availability between T0 and T1 (%)* | *+1 change in takeaway availability between T0 and T1 (%)* | *+2 change in takeaway availability between T0 and T1 (%)* |
| --- | --- | --- | --- | --- | --- |
| *Takeaway availability in 1 mile buffers around the home* | *96 (1%)* | *433 (6%)* | *6690 (88%)* | *317 (4%)* | *45 (1%)* |
| *Takeaway availability in 1 mile buffers around the workplace* | *97 (3%)* | *325 (10%)* | *2433 (72%)* | *470 (14%)* | *69 (2%)* |
| *Takeaway availability in 1 mile buffers around the home and workplace* | *137 (3%)* | *605 (12%)* | *3830 (75%)* | *431 (8%)* | *30 (1%)* |

Participants included in the study were: n=7,581 (home), n=3,394 (workplace) and n=5,093 (home and workplace)

Table S3. T0 characteristics of the current study sample and participants lost to follow-up in the Fenland cohort

|  | | Eligible participants in the longitudinal sample (N=7581) | | Participants lost to follow-up (N=4590) | |
| --- | --- | --- | --- | --- | --- |
|  | | N | Mean (SD), median; p25-p75, or N (%) | N | Mean (SD), median; p25-p75, or N (%) |
| Age, years (mean (SD)) | | 7581 | 49.3 (7.4) | 4590 | 47.5 (7.6) |
| Female (n (%)) | | 7581 | 3924 (51.8) | 4590 | 2631 (57.3) |
| Age at completion of full time education, years (mean (SD)) | | 7529 | 19.3 (4.4) | 4538 | 18.5 (4.2) |
| Current work status  (n (%)) | Work full time (> 30h/week) | 7532 | 5093 (67.6) | 4547 | 2879 (63.3) |
|  | Work part time (< 30h/week) |  | 1584 (21.0) |  | 994 (21.9) |
|  | Keeping house or carer |  | 383 (5.1) |  | 335 (7.4) |
|  | Wholly retired from work |  | 283 (3.8) |  | 153 (3.3) |
|  | Unemployed or waiting to start new job |  | 119 (1.6) |  | 92 (2.0) |
|  | Temporarily or permanently sick |  | 70 (0.9) |  | 94 (2.1) |
| Annual household income  (n (%)) | <£20,000 | 7419 | 833 (11.2) | 4409 | 800 (18.1) |
|  | £20,000-£39,999 |  | 2518 (33.9) |  | 1672 (37.9) |
|  | ≥£40,000 |  | 4068 (54.8) |  | 1937 (43.9) |
| Occupational social class  (n (%)) | Working class | 7267 | 1407 (19.4) | 4189 | 1214 (29.0) |
|  | Intermediate |  | 1228 (16.9) |  | 804 (19.2) |
|  | Professional |  | 4632 (63.7) |  | 2171 (51.8) |
| IMD score in quintiles  (mean (SD)) | 1 | 7195 | 3.8 (1.2) |  | 4.4 (1.3) |
|  | 2 |  | 7.4 (1.4) |  | 8.7 (1.6) |
|  | 3 |  | 12.2 (1.5) | 4306 | 14.3 (1.8) |
|  | 4 |  | 18.4 (2.1) |  | 20.2 (1.9) |
|  | 5 |  | 28.2 (6.1) |  | 30.6 (7.5) |
| Takeaway food consumption, serving/week (median (IQR)) | | 7557 | 1.5 (1.0 – 2.5) | 4560 | 1.5 (1.0 – 2.5) |
| Body fat percentage (mean (SD)) | | 7535 | 32.7 (8.4) | 4533 | 34.6 (9.1) |
| Fat mass index (mean (SD)) | | 7310 | 0.9 (0.3) | 4306 | 1.0 (0.4) |
| Body mass index (mean (SD)) | | 7580 | 25.9 (5.1) | 4582 | 26.8 (5.8) |

Table S4. Associations between takeaway availability in 1 mile buffers at T0, and change in BMI and FMI from T0 to T1 in the Fenland cohort

|  | BMI (kg/m^2^) | | FMI (fat mass in kg/m^2^) | |
| --- | --- | --- | --- | --- |
|  | β | 95%CI | β | 95%CI |
|  | Home environment^1^ | | | |
| No takeaways in 1 mile buffer | Ref | Ref | Ref | Ref |
| Some takeaways in 1 mile buffer | 0.03 | -0.09; 0.15 | 0.00 | -0.01; 0.01 |
| Most takeaways in 1 mile buffer | -0.11 | -0.28; 0.05 | -0.01 | -0.03; 0.00 |
|  | Workplace environment^2^ | | | |
| No takeaways in 1 mile buffer | Ref | Ref | Ref | Ref |
| Some takeaways in 1 mile buffer | -0.09 | -0.28; 0.09 | -0.01 | -0.02; 0.01 |
| Most takeaways in 1 mile buffer | -0.04 | -0.29; 0.21 | -0.01 | -0.03; 0.02 |
|  | Home and workplace environment^1^ | | | |
| No takeaways in 1 mile buffer | Ref | Ref | Ref | Ref |
| Some takeaways in 1 mile buffer | -0.14 | -0.33; 0.05 | -0.02 | -0.03; 0.00 |
| Most takeaways in 1 mile buffer | **-0.29** | **-0.52; -0.05** | **-0.03** | **-0.05; -0.01** |

Bold values are statistically significant

Abbreviations; Ref = Reference group, BMI = Body Mass Index, FMI = Fat Mass Index

Participants included in analyses was n=6722 for BMI and n=6487 for FMI in the home, n=3238 for BMI and n=3151 for FMI in the workplace and n=4477 for BMI and n=4340 for FMI in the home + workplace

^1^ Adjusted for baseline outcome, age, sex, age at completion of full time education, work status, annual household income, occupational social class, IMD quintiles and supermarket exposure as well as follow-up time and home relocation

^2^ Adjusted for baseline outcome, age, sex, age at completion of full time education, work status (only full-time or part-time), annual household income, occupational social class and supermarket exposure as well as follow-up time and home relocation

Table S5. Associations between takeaway exposure measures at T0, and change in takeaway consumption and body fat percentage from T0 to T1

|  | Takeaway consumption (g/day) | | Body fat (%) | |
| --- | --- | --- | --- | --- |
|  | β | 95%CI | β | 95%CI |
|  | Home environment^1^ | | | |
| No takeaways in 400m buffer | Ref | Ref | Ref | Ref |
| Some takeaways in 400m buffer | -0.76 | -2.46; 0.93 | -0.05 | -0.30; 0.20 |
| Most takeaways in 400m buffer | -2.01 | -3.85; -0.15 | -0.23 | -0.51; 0.05 |
| No takeaways in 800m buffer | Ref | Ref | Ref | Ref |
| Some takeaways in 800m buffer | -0.12 | -1.57; 1.34 | 0.15 | -0.07; 0.36 |
| Most takeaways in 800m buffer | -0.99 | -2.85; 0.88 | -0.25 | -0.52; 0.02 |
| No takeaways in 1 mile buffer | Ref | Ref | Ref | Ref |
| Some takeaways in 1 mile buffer | 1.04 | -0.48; 2.56 | 0.13 | -0.09; 0.36 |
| Most takeaways in 1 mile buffer | -0.28 | -2.44; 1.89 | -0.23 | -0.54; 0.09 |
| Furthest distance to nearest takeaway | Ref | Ref | Ref | Ref |
| Moderate distance to nearest takeaway | 0.27 | -1.30; 1.84 | 0.11 | -0.12; 0.33 |
| Closest distance to nearest takeaway | -0.89 | -2.44; 0.66 | 0.01 | -0.21; 0.24 |
| Furthest distance to five nearest takeaways | Ref | Ref | Ref | Ref |
| Moderate distance to five nearest takeaways | -0.25 | -1.93; 1.44 | 0.03 | -0.22; 0.28 |
| Closest distance to five nearest takeaways | -1.37 | -3.35; 0.61 | -0.14 | -0.43; 0.15 |
|  | Workplace environment^2^ | | | |
| No takeaways in 400m buffer | Ref | Ref | Ref | Ref |
| Some takeaways in 400m buffer | 0.05 | -2.03; 2.14 | 0.11 | -0.22; 0.43 |
| Most takeaways in 400m buffer | 0.18 | -2.34; 2.69 | 0.23 | -0.16; 0.63 |
| No takeaways in 800m buffer | Ref | Ref | Ref | Ref |
| Some takeaways in 800m buffer | 1.10 | -0.88; 3.09 | 0.28 | -0.03; 0.59 |
| Most takeaways in 800m buffer | 1.83 | -0.92; 4.58 | 0.25 | -0.18; 0.69 |
| No takeaways in 1 mile buffer | Ref | Ref | Ref | Ref |
| Some takeaways in 1 mile buffer | -0.15 | -2.36; 2.05 | -0.01 | -0.35; 0.34 |
| Most takeaways in 1 mile buffer | -0.15 | -3.12; 2.82 | 0.02 | -0.45; 0.49 |
| Furthest distance to nearest takeaway | Ref | Ref | Ref | Ref |
| Moderate distance to nearest takeaway | -0.44 | -2.45; 1.57 | 0.09 | -0.23; 0.40 |
| Closest distance to nearest takeaway | -0.99 | -3.07; 1.09 | 0.05 | -0.28; 0.38 |
| Furthest distance to five nearest takeaways | Ref | Ref | Ref | Ref |
| Moderate distance to five nearest takeaways | 1.02 | -1.15; 3.19 | 0.11 | -0.23; 0.45 |
| Closest distance to five nearest takeaways | 0.38 | -1.92; 2.68 | 0.04 | -0.32; 0.41 |
|  | Home and workplace environment^1^ | | | |
| No takeaways in 400m buffer | Ref | Ref | Ref | Ref |
| Some takeaways in 400m buffer | -0.44 | -2.09; 1.20 | -0.22 | -0.48; 0.05 |
| Most takeaways in 400m buffer | -2.27 | -4.14; -0.40 | -0.24 | -0.54; 0.07 |
| No takeaways in 800m buffer | Ref | Ref | Ref | Ref |
| Some takeaways in 800m buffer | 0.79 | -1.00; 2.59 | -0.03 | -0.33; 0.26 |
| Most takeaways in 800m buffer | 0.88 | -1.41; 3.17 | -0.20 | -0.57; 0.17 |
| No takeaways in 1 mile buffer | Ref | Ref | Ref | Ref |
| Some takeaways in 1 mile buffer | 0.95 | -1.21; 3.12 | -0.31 | -0.66; 0.04 |
| Most takeaways in 1 mile buffer | 1.91 | -0.81; 4.64 | **-0.68** | **-1.12; -0.24** |
| Furthest distance to nearest takeaway | Ref | Ref | Ref | Ref |
| Moderate distance to nearest takeaway | 0.12 | -1.54; 1.78 | -0.11 | -0.39; 0.17 |
| Closest distance to nearest takeaway | -0.70 | -2.38; 0.98 | -0.17 | -0.46; 0.12 |
| Furthest distance to five nearest takeaways | Ref | Ref | Ref | Ref |
| Moderate distance to five nearest takeaways | 1.32 | -0.39; 3.02 | -0.04 | -0.33; 0.26 |
| Closest distance to five nearest takeaways | 0.88 | -1.06; 2.82 | -0.11 | -0.46; 0.23 |

Bold values are statistically significant

Abbreviations; Ref = Reference group

In home environment, n=6670 and n=6640 participants were included for takeaway consumption and body fat percentage, respectively. In the work environment, n=3216 and n=3198 participants were included, and in the combined environment n=4445 and n=4417 participants were included for takeaway consumption and body fat percentage, respectively.

^1^ Adjusted for baseline outcome, age, sex, age at completion of full time education, work status, annual household income, occupational social class, IMD quintiles and supermarket exposure as well as follow-up time and home relocation

^2^ Adjusted for baseline outcome, age, sex, age at completion of full time education, work status (only full-time or part-time), annual household income, occupational social class and supermarket exposure as well as follow-up time and home relocation

Table S6. Cross-sectional associations between takeaway availability in 1 mile buffers, and takeaway consumption and body fat percentage separately at T0 and T1 in the Fenland cohort

|  | Takeaway consumption (g/day) | | | | Body fat (%) | | | |  |  |
| --- | --- | --- | --- | --- | --- | --- | --- | --- | --- | --- |
|  | T0^1^ | | T1^2^ | | T0^1^ | | T1^2^ | |  |  |
|  | β | 95%CI | β | 95%CI | β | 95%CI | β | 95%CI |  |  |
|  | Home environment^3^ | | | | | | | |  |  |
| No takeaways in 1 mile buffer | Ref | Ref | Ref | Ref | Ref | Ref | Ref | Ref |  |  |
| Some takeaways in 1 mile buffer | 1.51 | -0.21; 3.23 | **1.77** | **0.14; 3.40** | **0.41** | **0.00; 0.82** | **0.52** | **0.11; 0.92** |  |  |
| Most takeaways in 1 mile buffer | 0.95 | -1.49; 3.39 | -0.49 | -2.68; 1.70 | 0.55 | -0.03; 1.13 | -0.01 | -0.50; 0.63 |  |  |
|  | Workplace environment^4^ | | | | | | | |  | Work environment^4^ |
| No takeaways in 1 mile buffer | Ref | Ref | Ref | Ref | Ref | Ref | Ref | Ref |  |  |
| Some takeaways in 1 mile buffer | 0.61 | -2.03; 3.25 | -2.42 | -4.96; 0.13 | **0.90** | **0.25; 1.55** | 0.41 | -0.25; 1.07 |  |  |
| Most takeaways in 1 mile buffer | **5.40** | **1.86; 8.94** | 1.31 | -1.82; 4.43 | **1.68** | **0.81; 2.55** | **1.22** | **0.41; 2.03** |  |  |
|  | Home and workplace environment^3^ | | | | | | | |  | Home + work environment^3^ |
| No takeaways in 1 mile buffer | Ref | Ref | Ref | Ref | Ref | Ref | Ref | Ref |  |  |
| Some takeaways in 1 mile buffer | 0.54 | -2.12; 3.20 | 1.51 | -0.64; 3.67 | **1.08** | **0.43; 1.73** | **0.80** | **0.22; 1.38** |  |  |
| Most takeaways in 1 mile buffer | **3.63** | **0.28; 6.98** | 1.86 | -0.84; 4.56 | **1.30** | **0.48; 2.11** | 0.70 | -0.02; 1.43 |  |  |

Bold values are statistically significant

^1^ T0: N=6702 for takeaway consumption and N=6681 for body fat percentage in home environment, N=3246 for takeaway consumption and N=3228 for body fat percentage in work environment, and N=4534 for takeaway consumption and N=4508 for body fat percentage in home + work environment

^2^ T1: N=7193 for takeaway consumption and N=7174 for body fat percentage in home environment, N=3308 for takeaway consumption and N=3304 for body fat percentage in work environment, and N=4805 for takeaway consumption and N=4791 for body fat percentage in home + work environment

^3^ Adjusted for age, sex, age at completion of full time education, work status, annual household income, occupational social class, IMD quintiles, supermarket exposure

^4^ Adjusted for age, sex, age at completion of full time education, work status (only full-time or part-time), annual household income, occupational social class and supermarket exposure


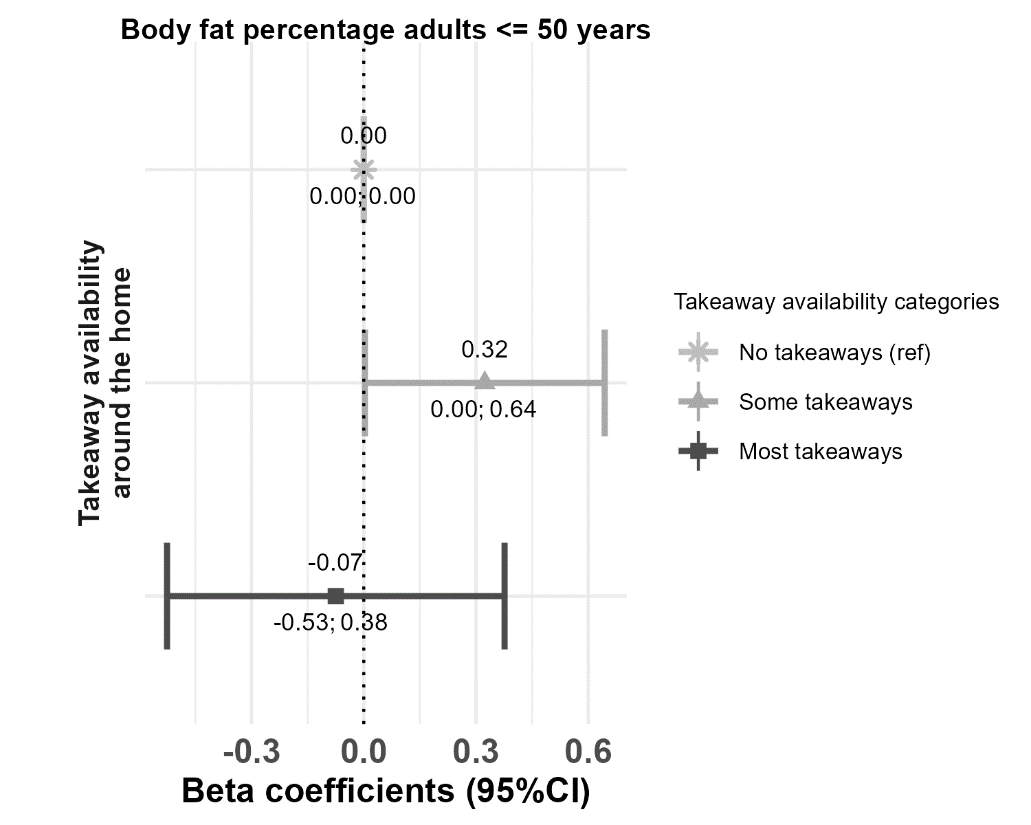

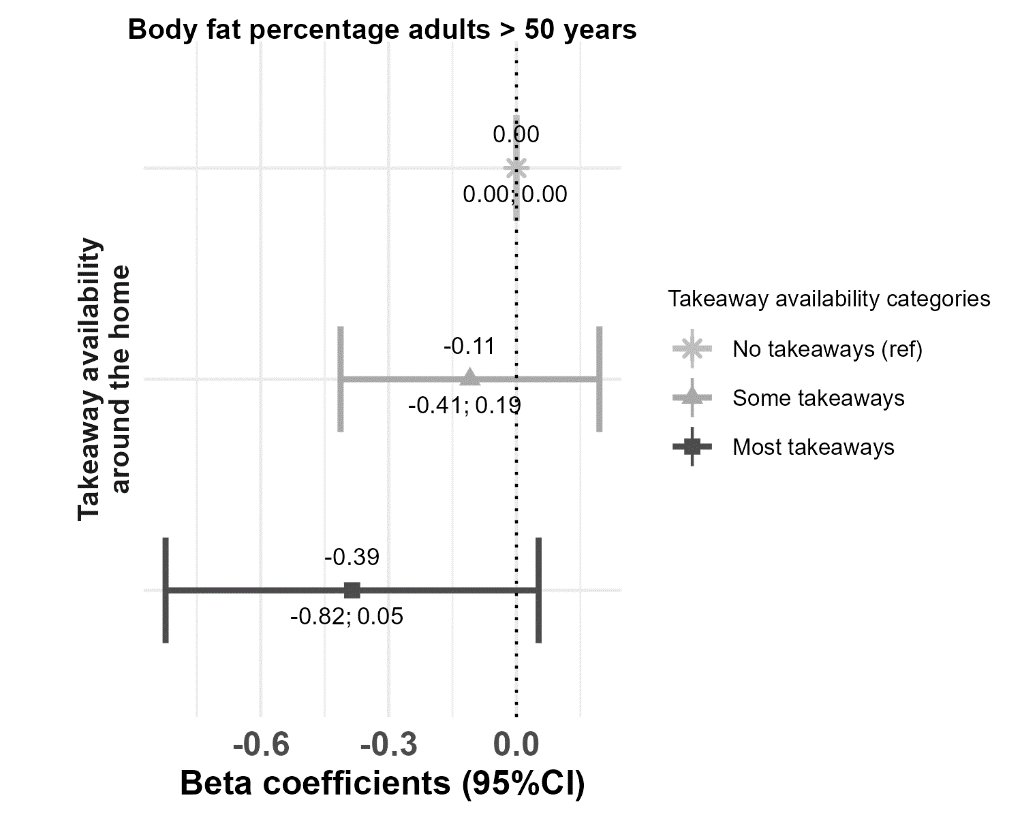


Figure S1. Associations between takeaway availability around the home at T0 and change in body fat percentage from T0 to T1 for younger (n= 3581) and older adults (n= 3059). *Beta-coefficients represent the difference in mean outcome per 1 extra takeaway adjusted for outcome at T0, follow-up time, age, sex, age at completion of full time education, work status, annual household income, occupational social class, IMD quintiles, supermarket availability and home relocation.*
